# Supplementary material for: Infective Endocarditis—Predictors of In-Hospital Mortality, 17 Years, Single-Center Experience in Bulgaria
Source: Microorganisms. 2024 Sep 21;12(9):1919. doi: 10.3390/microorganisms12091919 (PMC11434097; doi:10.3390/microorganisms12091919)
Supplement: Supplementary file 1 [file microorganisms-12-01919-s001.zip › microorganisms-3203458-supplementary.pdf]

Supplementary Table S1. Echocardiographic results.

Variables	Total IE cases 2005 – 2021 n=270	IE Non-Survivors n=67	IE Survivors n=203	p=value
TTE	270 (100)	67 (100)	203 (100)	N/A
TTE + TOE	97 (35.9)	11 (16.4)	86 (42.4)	0.000*
Valve location, n (%)				
AV	121 (44.8)	28 (41.8)	93 (45.8)	0.568*
MV	74 (27.4)	17 (25.4)	58 (28.6)	0.612*
TV	26 (9.6)	5 (7.5)	21 (1p0.3)	0.500*
PV	1 (0.4)	1 (1.5)	0 (0.0)	0.080*
Bivalve IE	45 (16.7)	14 (20.8)	31 (15.3)	0.287*
AV - MV	37 (13.7)	10 (14.9)	27 (13.3)	0.741*
AV - TV	4 (1.5)	3 (4.5)	1 (0.5)	0.019*
MV - TV	4 (1.5)	1 (1.5)	3 (1.5)	1*
CDRIE	2 (0.74)	2 (3.0)	0 (0)	-
Vegetations, n (%)	226 (83.7)	56 (83.6)	180 (83.7)	0.985*
< 10 mm	153 (56.7)	37 (55.2)	116 (57.1)	0.786*
10-15 mm	38 (14.1)	9 (13.4)	29 (14.3)	0.854*
> 15 mm	35 (13.0)	10 (14.9)	25 (12.3)	0.583*
Perivalvular abscess, n (%)	8 (3.0)	1 (1.5)	7 (3.4)	0.424*
Chordal rupture, n (%)	5 (3.3)	1 (1.5)	4 (1.97)	0.805*
EF %. медиана (IQR)	60 (54-68)	55 (51-66)	62 (55-68)	0.001†
Valve obstruction, n (%)	32 (11.9)	10 (14.9)	22 (10.8)	0.369*
Aortic regurgitation, n (%)	146 (54)	39 (58.2)	107 (52.7)	0.433*
Mild - moderate	79 (29.3)	25 (37.3)	54 (26.6)	0.095*
Severe	67 (24.8)	14 (20.9)	53 (26.1)	0.393*
Mitral regurgitation, n (%)	115 (42.6)	33 (49.3)	82 (40.4)	0.202*
Mild - moderate	64 (23.7)	18 (26.9)	46 (22.6)	0.473*
Severe	51 (18.9)	15 (22.4)	36 (17.8)	0.405*
Tricuspid regurgitation, n (%)	36 (13.3)	8 (11,9)	28 (13.7)	0.704*
Mild - moderate	16 (6.0)	1 (1.5)	15 (7.3)	0.080*
Severe	20 (7.4)	7 (10.5)	13 (6.4)	0.267*

*z-test; †Mann-Whitney U Test; ; TTE—transthoracic echocardiography; TOE—transesophageal echocardiography; AV—aortic valve; MV—mitral valve; TV—tricuspid valve; PV- pulmonic valve; CDRIE—cardiac device related IE; EF—ejection fraction.

Supplementary Table S2. Microbiological agent.

Microbiological agent, n (%)	Total IE cases 2005 – 2021 n=270	IE Non survivors n-67	Survivors n-203	p-value*
Negative hemoculture	111 (41.1)	24 (35.8)	87 (42.8)	0.271
Staphylococci spp.	89 (33.0)	21 (31.3)	68 (33.5)	0.740
Staphylococcus aureus	51 (18.9)	12 (17.9)	39 (19.2)	0.814
Staphylococcus CoNS	38 (14.1)	9 (13.4)	29 (14.3)	0.854
Streptococci spp.	21 (7.8)	3 (4.5)	18 (7.0)	0.468
Streptococcus viridans	9 (3.0)	2 (3.0)	7 (3.0)	1.000
Streptococcus beta-hemolyticus	2 (0.4)	0 (0.0)	2 (0.5)	0.562
Streptococcus alfa hemolyticus	6 (2.2)	1 (1.5)	5 (2.5)	0.632
Streptococci -други	4 (1.5)	0 (0.0)	4 (2.0)	0.244
Enterococci spp.	25 (9.3)	8 (11.9)	17 (8.4)	0.392
Enterococcus species	1 (0.4)	0 (0.0)	1 (0.5)	0.562
Enterococus faecalis	23 (8.5)	8 (11.9)	15 (7.4)	0.253
Enterococcus durans	1 (0.4)	0 (0.0)	1 (0.5)	0.562
Gram negative (non-HACEK) spp.	19 (7.0)	10 (14.9)	9 (4.5)	0.004
Pseudomonas aeruginosa	2 (0.7)	1 (1.5)	1 (0.5)	0.410
Escherichia coli	9 (3.4)	5 (7.4)	4 (2.0)	0.030
Enterobacter cloacae	1 (0.4)	0 (0.0)	1 (0.5)	0.562
Klebsiella pneumoniae	3 (1.1)	1 (1.5)	2 (1.0)	0.736
Serratia marcescens	4 (1.5)	3 (4.5)	1 (0.5)	0.019
Others	5 (1.9)	2 (3.0)	3 (1.5)	0.432
Candida spp	3 (1.1)	1 (1.5)	2 (1.0)	0.736
Erysipelothix rhusiopathiae	1 (0.4)	0 (0.0)	1 (0.5)	0.562
Brevibacterium casei	1 (0.4)	1 (1.5)	0 (0.0)	0.080

* z – test; CoNS—coagulase-negative staphylococcus; non-HACEK- (Hemophilus species, Actinobacillus, Cardiobacterium, Eikenella, or Kingella).
